# Long-term survival of an elderly female with metastatic colon cancer after treated with capecitabine monotherapy: A case report

**DOI:** 10.1097/MD.0000000000014326

**Published:** 2019-02-01

**Authors:** Jin Liu, Yao Wang, Haiping Jiang, Xiongfei Yu, Nong Xu

**Affiliations:** a Department of Medical Oncology, First Affiliated Hospital, School of Medicine, Zhejiang University, 79# Qingchun Road; b Department of Medical Oncology, Fujian Medical University Union Hospital, 29# Xinquan Road, Fuzhou; c Department of Surgical Oncology, First Affiliated Hospital, College of Medicine, Zhejiang University, 79# Qingchun Road, Hangzhou, China.

**Keywords:** capecitabine, elderly patient, metastatic colorectal cancer

## Abstract

**Rationale:**

Colorectal cancer (CRC) is one of the most common cancers all over the world, and approximately 70% of the newly diagnosed patients are over 65 years old. Due to the aging of society, there will be more and more elderly patients of CRC in the future. Treatment of CRC in elderly patients is much more challenging thanks to multiple factors including disabling comorbidities as well as declines in organs function, especially in advanced or metastatic settings.

**Patient concerns:**

An 82-year-old female without history of disease was admitted to the emergency room because of abdominal pain in December 2009. A computed tomography (CT) scan of the abdomen was performed immediately, which revealed bowel obstruction.

**Diagnoses:**

The histopathological examination of the resected specimen confirmed well-to-moderately differentiated colonic adenocarcinoma with a stage of IIIB (T3N1M0) based on the NCCN tumor-node-metastasis (TNM) classification system.

**Interventions:**

The patient underwent emergency surgery and rejected adjuvant chemotherapy, but was diagnosed with multiple liver metastases 3 months later. Then she received capecitabine monotherapy as first-line treatment.

**Outcomes:**

The efficacy achieved complete response (CR) when 8 cycles were completed and the agent was continued as maintenance treatment until totally 16 cycles were completed. Up to the latest follow-up, the disease remained CR and the progression-free survival (PFS) has achieved approximately 87 months. She is still alive and has good quality of life.

**Lessons:**

Capecitabine monotherapy may be an effective treatment in advanced or metastatic colorectal cancer (mCRC) for elderly patients.

## 1 Introduction

Colorectal cancer, the second leading cause of cancer-related deaths, remains a major malignant neoplasm in the USA and worldwide with a median age at diagnosis of 71 years, and 40% of cases are over 75 years old at diagnosis.^[1]^ Furthermore, there are about 20% of newly diagnosed patients are in metastatic settings.^[2]^ Predominant advances have been made in treating mCRC over the past decades and systemic chemotherapy has long been the mainstay. However, owing to their geriatric characteristics such as poor performance status, multiple comorbidities, function impairment of important organs and poor tolerance to toxicity of chemotherapy, most of the elderly patients can’t be treated according to the standard guidelines applied to their younger counterparts. Additionally, they are often excluded or underrepresented from most of clinical trials.^[3]^ Therefore, treating these senile patients with mCRC effectively and safely still remains an unsolved issue. Capecitabine, the oral drug which was initially developed to improve tolerability and to reduce non-tumor cytotoxicity, is a tumor-activated 5-FU prodrug.^[4]^ Several studies have demonstrated that capecitabine monotherapy is at least as effective as infusional 5-FU in advanced mCRC and may provide an alternative option for elderly mCRC patients who are frail or ineligible for combination chemotherapy or aggressive chemotherapy.^[5,6]^ Herein, we report an elderly female with metastatic colon cancer who was treated with capecitabine monotherapy and obtained long PFS, as well as long-term survival. To the best of our knowledge, few cases like this have been reported before.

## 2 Case report

**Figure 1:**
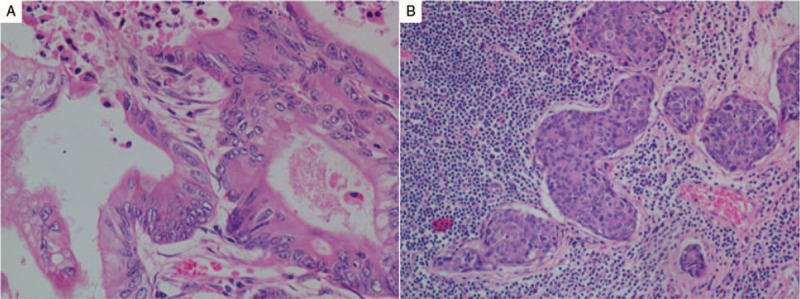
A. Pathologic result showed well-to-moderately differentiated adenocarcinoma (×400). B. Pathologic result showed cancer embolus (×200).

**Figure 2:**
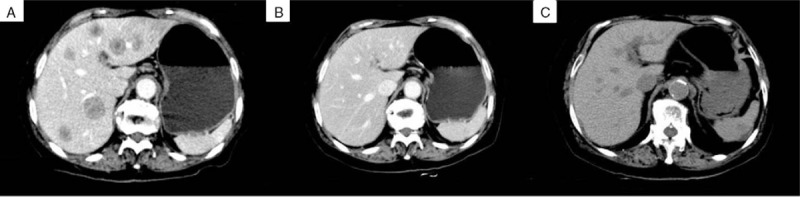
A. Base-line CT scan of the liver in March 2010 before first-line chemotherapy: multiple occupied lesions in liver. B. Image of achieving CR in October 2010: the metastatic liver adenocarcinoma completely disappeared. C. Image of the latest follow-up in January 2018: no new metastatic lesions occurred (non-contrast enhanced CT was made due to her high level of creatinine).

**Figure 3:**
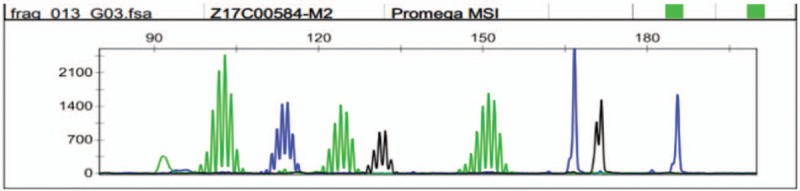
No site length variation was found and refer to the MSI criteria, the sample submitted was MSS. MSI = microsatellites instability, MSS = microsatellite stable.

An 82-year-old female without history of disease was admitted to the emergency room because of abdominal pain in December 2009. Left colonic obstruction was revealed by the CT scan of the abdomen. She underwent left hemicolectomy subsequently and pathological examination displayed well-to-moderately differentiated adenocarcinoma (Fig. 1A) with serosal invasion, lymph node metastases (2/16) and lymph vessel tumor emboli (Fig. 1B). The stage was IIIB (T3N1M0) based on the NCCN TNM classification system. She refused adjuvant chemotherapy but developed multiple liver metastases (Fig. 2A) 3 months later. The carcinoembryonic antigen (CEA) also increased to 9.5 ng/ml. Physical examination indicated no significant findings and her ECOG performance status was 0. Considering her old age, capecitabine monotherapy (1250 mg/m^2^ twice daily on days 1–14, every 3 weeks) was administered as first-line treatment on March 10, 2010. The liver metastases shrunk by 20% and CEA sharply decreased to 3.2 ng/ml when 2 cycles were completed. After 8 cycles, the metastases completely disappeared (Fig. 2B). The regimen was continued until 12 cycles were completed and another 4 cycles were followed as maintenance chemotherapy. During the whole course, the patient suffered leucopenia of grade 1 and hand-foot syndrome of grade 1, which were mild and controllable. No evidence of recurrence or metastasis was observed and CEA was also within normal range during her routine review. Considering the inspiring long PFS observed, we conducted a genetic test in October 2013, which exposed BRAF V600E mutation and KRAS wild-type, low mRNA expression of thymidylate synthase (TS), low mRNA expression of PTEN. The microsatellite instability (MSI) status was microsatellite stable (MSS) (Fig. 3). UP to the latest follow-up in January 2018, no evidence of recurrence or metastasis was observed in liver (Fig. 1C) and the PFS has reached up 87 months. At present, the female is still alive and enjoying good quality of life.

## 3 Discussion

Currently, regimens based on fluorouracil (5-FU or capecitabine) with oxaliplatin or irinotecan plus the targeted drugs bevacizumab, cetuximab or panitumumab are standard first-line treatment for mCRC patients with RAS and BRAF wild-type. However, these may not work out for all elderly patients owing to their geriatric characteristics. Individualized treatment of elderly patients with mCRC is challenging and more concentration should be focused on comprehensive assessment of elderly patients, such as estimation in life-expectancy, risk of cancer-related morbidity, the conditions that could interfere in the cancer treatment and tolerance, particularly their quality of life.^[3]^

Agents that could be used to treat the elderly mCRC patients are reatively limited when compared to those applied in younger mCRC patients. A retrospective analysis comparing the safety and efficacy of oxaliplatin-contained doublet with 5-FU alone uncovered that grade ≥3 hematologic toxicity including neutropenia and thrombocytopenia were much severer in older patients, suggesting that oxaliplatin-contained doublet may only be suitable for selected elderly patients.^[7]^ FFCD2001–02 study compared the 5-FU-based regimens with or without irinotecan as first-line chemotherapy in over 75-year-old mCRC patients. The results demonstrated that the addition of irinotecan improved neither PFS nor overall survival (OS), but toxicities.^[8,9]^ Capecitabine is an orally administered anti-metabolite fluoropyrimidine deoxynucleotide carbamate that can be converted to 5-FU in the body to exert anti-tumor effects by inhibiting cell division and interfering synthesis of RNA and proteins. A randomized phase III trial evaluating the efficacy between regimens FOLFOX4 (leucovorin, 5-FU plus oxaliplatin) and XELOX (capecitabine plus oxaliplatin) showed that no significant differences were observed in objective response rate (ORR), PFS and OS, respectively.^[10]^ As for side effects, patients using FOLFOX4 suffered more grade 3/4 myelosuppression, while those with XELOX experienced more diarrheas. Another study also manifested no significant differences in ORR, PFS, and OS between the regimens FOLFIRI (5-FU, leucovorin plus irinotecan) and XELIRI (capecitabine plus irinotecan).^[11]^ All of these studies above suggested that oral capecitabine was equivalent to infusional 5-FU in treating mCRC. Moreover, the oral administration of capecitabine permits dosage flexibility and prolonged drug exposure while avoiding the inconvenience of infusion pumps or vascular devices and associated complications. Its home-based administration also greatly reduces the time of hospitalization and is more appropriate for those reluctant to be hospitalized. Bevacizumab, the recombinant humanized monoclonal antibody against vascular endothelial growth factor (VEGF), has been a standard care of mCRC based on prior studies demonstrating that median PFS, median OS, and ORR were significantly improved in patients treated with capecitabine plus bevacizumab compared with those using capecitabine alone.^[12]^ This made the capecitabine plus bevacizumab regimen an alternative for fit elderly mCRC patients in first-line treatment.^[13]^ Capecitabine can also be used as maintenance therapy at a lower dose for those patients.^[14]^ In our case, we administrated capecitabine alone as the patient's palliative treatment due to her old age and unwillingness to be hospitalized. The reason for the absence of bevacizumab was that the biologic agent was beyond to China's medical insurance system at that time and the patient could not afford the high cost.

With the development of molecular biology, genetic test has been a significant means to assist clinical treatment decision-making. In the presented case, we conducted genetic tests after the PFS, but in clinical practice this should be performed in advance to provide information for clinical decision-making as driver gene mutation status is closely associated with treatment options. KRAS plays substantial role in many molecular pathways and approximately 33% of CRC have point mutations in exon 2 or exon 3 of KRAS. Those mutations cause permanent activation of the RAS (RAS/RAF/MAPK) pathway and predict resistance to anti-EGFR therapy.^[15,16]^ As the direct downstream target of KRAS in the RAS pathway, BRAF mutated frequently in CRC.^[17]^ Approximately 8% of CRC carry the BRAF V600E mutation, which determines resistance to the anti-EGFR therapy and is associated with poor prognosis in the MSS CRC.^[18,19]^ DNA mismatch repair (MMR) system controls the newly synthesized DNA strands and corrects polymerase misincorporation events. A germ line mutation of 1 of the 4 MMR genes (MSH2, MLH1, MSH6, and PMS2) leads to MSI,^[20]^ and about 15% of CRC displays DNA MMR deficiency and the high-level microsatellite instability (MSI-H) phenotype. MSI-H CRC is characterized by a distinct clinical behavior and has better prognosis than their MSS counterparts.^[21]^ PTEN is a tumor suppressor gene, inactivation of which causes deregulation of the PI3K pathway and loss of which has been associated with aggressive CRC and predicts nonresponse to cetuximab.^[22]^ TS is the target enzyme for fluorouracil. Studies on mCRC have demonstrated that high TS levels are correlated with resistance to fluoropyrimidine,^[23]^ while low expression of TS mRNA predicts favorable response to fluorouracil-based chemotherapy and long-term survival in CRC patients.^[24]^

Our patient rapidly developed liver metastases after surgery, except for emergency surgery^[25]^ and absence of adjuvant chemotherapy, factors consisting of BRAF V600E mutation and MSS status may also contribute to the aggressiveness. Fortunately, the patient achieved an exciting efficacy of CR when 8 cycles of capecitabine were completed and obtained long-term survival, which was encouraging, as few patients using capecitabine alone had obtained such great efficacy, long-term survival were also rare. There were only 4 out of 603 advanced CRC patients treated with capecitabine alone achieved CR in 2 phase III randomized clinical trials.^[5,6]^ And in AVEX study, only 2 out of 140 patients treated with capecitabine achieved CR.^[12]^ Maybe factors including mCRC with left-sided primary,^[26]^ free of comorbidities and low mRNA expression of PTEN contribute to the great response to capecitabine and long-term survival.

The most common side effect of capecitabine is grade 3/4 hand-foot syndrome. Capecitabine may also cause diarrhea and mucositis but less grade 3/4 myelosuppression occurs when compared to infusional 5-FU.^[27]^ Our patient encountered grade 1 leucopenia and grade 1 hand-foot syndrome, which were tolerated and manageable.

Our case suggests that capecitabine monotherapy may be an effective and well tolerated treatment for elderly patients with mCRC in first-line and maintenance treatment. Comprehensive assessment and genetic test should be made ahead of the initial treatment to approach individualized medical care and maximum benefits.

## Author contributions

**Conceptualization:** Jin Liu, Nong Xu.

**Data curation:** Yao Wang.

**Formal analysis:** Haiping Jiang.

**Funding acquisition:** Xiongfei Yu.

**Supervision:** Haiping Jiang.

**Writing – original draft:** Jin Liu.

**Writing – review & editing:** Nong Xu.
